# The Dual-Edged Sword Effect of Human–Robot Collaboration on Migrant Workers’ Well-Being: Evidence from China

**DOI:** 10.3390/bs16040526

**Published:** 2026-04-01

**Authors:** Ruonan Wang, Guangsheng Zhang

**Affiliations:** 1 School of Business, Shenyang University, Shenyang 110044, China; 2 School of Business, Liaoning University, Shenyang 110036, China

**Keywords:** perceived human-robot collaboration, migrant workers’ well-being, perceived decent work, job replacement anxiety, perceived organizational support

## Abstract

Migrant workers are a unique group under China’s urban–rural dual system, and improving their well-being is an intrinsic requirement for achieving common prosperity. In recent years, human–robot collaboration has come forth as a novel work paradigm. Comprehending the influence of human–robot collaboration on the well-being of migrant workers is a vital yet insufficiently investigated matter. With the conservation of resources theory as an analytical framework, this study empirically analyzes data from a two-stage survey of 382 migrant workers in Chinese manufacturing enterprises. The findings suggest that perceived human–robot collaboration can positively influence migrant workers’ well-being by facilitating perceived decent work, while also negatively affecting their well-being by increasing job replacement anxiety. Perceived organizational support plays a positive moderating role in two distinct aspects: on the one hand, the relationship between perceived human–robot collaboration and perceived decent work; on the other hand, the mediating effect through which perceived decent work connects perceived human–robot collaboration with the well-being of migrant workers. Conversely, perceived organizational support exerts a negative moderating effect on the association between perceived human–robot collaboration and job replacement anxiety, while job replacement anxiety functions as a mediator in the relationship linking perceived human–robot collaboration to migrant workers’ well-being. This study helps understand how human–robot collaboration in manufacturing enterprises affects the well-being of migrant workers.

## 1. Introduction

Currently, China has entered the era of intelligence, with a large number of artificial intelligence devices entering the workplace, evolving from auxiliary tools to important work partners. Based on statistics from the International Federation of Robotics, the volume of industrial robots installed across China has shown an overall upward trend in recent years. Starting in 2021, China’s installed base of industrial robots has surpassed the combined total of such installations in all other countries globally. While the deployment of robots has resulted in a growing replacement of workers engaged in routine and repetitive job responsibilities, robots remain incapable of independently accomplishing excessively complex tasks. As a result, partial “robot replacement” and overall “human–robot symbiosis” have become the mainstream development direction. The collaborative cooperation between human employees and collaborative robots has emerged as a new work model (J. M. Li et al., 2025).

Migrant workers are a unique group formed under China’s urban–rural dual system. Enhancing their well-being is of significant importance for accelerating the development of new urbanization and achieving common prosperity for all people. The “Migrant Worker Monitoring Survey Report” indicates that in 2024, the total number of migrant workers in China was 299.73 million, an increase of 0.7% compared to the previous year. As an important part of the industrial workforce, migrant workers have been pivotal in boosting China’s economic prosperity (Cai, 2018). However, compared to urban workers, migrant workers, due to their relatively low levels of human capital, mostly engage in high-intensity and high-risk jobs on the front lines. Low income, poor treatment, lack of respect, and low sense of presence are prominent issues faced by migrant workers, resulting in relatively low levels of well-being (Knight & Gunatilaka, 2010). Improving the migrant workers’ well-being has become a pressing issue that requires urgent attention.

For migrant workers engaged in human–robot collaboration, robots can replace them in performing repetitive and hazardous tasks, which helps improve the working environment and reduce labor intensity. In addition, human–robot collaboration can enhance career development capabilities through training and other means, thereby facilitating perceived decent work and promoting an increase in well-being. However, human–robot collaboration also raises the skill requirements for employees (Acemoglu & Restrepo, 2018). Compared to general workers, migrant workers, due to their relatively lower levels of human capital, may face greater difficulties and challenges during the human–robot collaboration process. This increases their likelihood of being replaced and negatively impacts their well-being. Thus, does human–robot collaboration enhance or reduce the well-being of migrant workers? What is the underlying mechanism? How can we further strengthen the positive impact of human–robot collaboration on migrant workers’ well-being and weaken its negative impact? Addressing these questions is crucial for understanding how human–robot collaboration affects migrant workers’ well-being and how it can be leveraged to improve their well-being.

Existing research predominantly explores the impact of robot applications on migrant workers’ urban employment and settlement intentions from a macro-level perspective (Ghodsi et al., 2024; Zhou et al., 2024). At the micro-level, the effects of human–robot collaboration on employees’ psychological and behavioral aspects are mainly studied from the perspective of general employees (Gao et al., 2023; Loureiro et al., 2023; Yin et al., 2024), with a lack of targeted research focusing on migrant workers. In response to this gap, this paper, based on Resource Conservation Theory, constructs a dual-edged sword effect model to examine the impact of perceived human–robot collaboration on the well-being of migrant workers. Empirical testing is conducted using survey data from migrant workers employed in manufacturing enterprises. This study aims to reveal the underlying mechanisms by which human–robot collaboration affects the well-being of migrant workers, identify the boundary conditions of these effects, and provide theoretical insights for further strengthening the positive influence of human–robot collaboration on migrant workers’ well-being while mitigating its negative effects.

## 2. Literature Review

Existing research on the factors influencing migrant workers’ well-being has explored these influences from individual, work, and social levels in depth. It is widely acknowledged that individual characteristics such as gender, age, marital status, education level, health status, self-confidence, and extraversion; as well as work-related factors such as wage income, working hours, labor protection, career development opportunities, and job dignity; along with social factors like social capital, social integration, and social support, all exert a significant influence on the well-being of migrant workers (Chu & Hail, 2014; Zhou et al., 2025; Agyeiwaah et al., 2024). Given that employment is the primary reason for migrant workers’ migration to cities, work characteristics have an especially profound impact on their well-being. Nonetheless, current studies mainly concentrate on traditional contexts and rarely address the impact of new work models in the digital intelligence era on migrant workers’ well-being.

As the application of robots in the workplace expands, academic attention has gradually shifted toward the consequences of human–robot collaboration for employees’ psychological and behavioral outcomes, though no consensus has been reached. Scholars who adopt a positive perspective argue that human–robot collaboration helps to expand the resources and psychological safety of employees in their work; enhances psychological empowerment (Gao et al., 2023); satisfies needs for autonomy, competence, and relatedness (Yang & Gao, 2025); and increases overall well-being (Shaikh et al., 2023; Loureiro et al., 2023), proactive behavior (J. M. Li et al., 2025), and service performance (Tan et al., 2025). Conversely, scholars who adopt a negative perspective contend that human–robot collaboration increases role ambiguity and feelings of loneliness (Tang et al., 2022; Tang et al., 2023); diminishes workers’ sense of freedom and meaning; and heightens cognitive load, external surveillance, and insecurity (Nazareno & Schiff, 2021), leading to negative emotions that affect job satisfaction and well-being (N. C. Liu et al., 2023). Furthermore, several studies indicate that human–robot collaboration can exert both favorable and adverse influences on employees’ task performance and innovative behaviors (Tang et al., 2022; C. He et al., 2023; Yin et al., 2024; Yang & Wu, 2026). In contrast, existing research examining how human–robot collaboration affects employee well-being is still mostly limited to one-sided analyses that focus solely on either its positive impacts or its negative consequences. Furthermore, in studies investigating the psychological and behavioral impacts of human–robot collaboration, existing literature primarily examines factors influencing the effectiveness of such collaboration, including individual traits and organizational characteristics—for example, individual traits such as conscientiousness (Tang et al., 2022), future orientation (Liang et al., 2022), and self-affirmation (Yam et al., 2022), along with organizational factors including authoritarian organizational culture (Lingmont & Alexiou, 2020) and transformational leadership (Wijayati et al., 2022). Unfortunately, academic research on the ramifications of human–robot collaboration for employee well-being has primarily focused on individual-level determinants like skill flexibility and attachment anxiety (Tang et al., 2023; N. C. Liu et al., 2023).

As an important component of corporate human resources, the impact of human–robot collaboration on the well-being of migrant workers is a crucial issue that deserves attention. However, due to the relatively disadvantaged position of migrant workers in the labor market, academic discussions have mostly focused on the macro-level effects of robot applications on their urban employment or settlement intentions (Ghodsi et al., 2024; Zhou et al., 2024). Notably, insufficient attention has been paid to the psychological and behavioral challenges faced by migrant workers involved in human–robot collaboration. Consequently, there is an urgent need to carry out in-depth investigations into the connection between human–robot collaboration and the well-being of migrant workers.

## 3. Theoretical Analysis and Research Hypotheses

### 3.1. The Positive Effect of Perceived Human–Robot Collaboration on the Well-Being of Migrant Workers Through Perceived Decent Work

Decent work refers to the level of dignity of migrant workers’ jobs, reflecting the quality of their work. First, human–robot collaboration helps improve the dignity of migrant workers’ work rewards. On one hand, human–robot collaboration enhances the complementarity between labor and technology, which can improve production efficiency (Frey & Osborne, 2017) and output per unit of time. Under sound corporate human resource policies and labor protection conditions, the higher productivity and profitability driven by human–robot collaboration can be translated into higher wage income for migrant workers. On the other hand, the improvement in production efficiency brought by human–robot collaboration helps enhance corporate profitability, which in turn motivates firms to expand production scale and further upgrade production efficiency (Acemoglu & Restrepo, 2020). This not only leads to higher wage income for migrant workers but also improves the dignity of work rewards. Secondly, human–robot collaboration helps improve the dignity of migrant workers’ working hours. In traditional contexts, migrant workers often work overtime to earn higher wages, which negatively impacts their physical and mental health. Human–robot collaboration automates complex manual labor, significantly improving production efficiency and reducing labor time (K. Liu et al., 2025), thus enhancing the dignity of migrant workers’ working hours. Thirdly, human–robot collaboration helps improve the dignity of migrant workers’ career development. The human–robot collaborative work model requires higher job skills from employees (Acemoglu & Restrepo, 2018). To meet the skill demands of human–robot collaboration, migrant workers often enhance their work capabilities by participating in skill training and actively learning, thereby improving their vocational skills (Barrett et al., 2012; Gray & Suri, 2017) and enhancing the dignity of their career development. Lastly, human–robot collaboration can improve the dignity of migrant workers’ career recognition. In traditional settings, migrant workers engage in strenuous, exhausting work on the front lines, with marginalized positions and low professional recognition (Wong et al., 2008). Under the human–robot collaboration model, robots can replace migrant workers in performing repetitive, dangerous and physically demanding tasks, allowing migrant workers to engage in more creative work. This not only reduces their labor intensity but also enhances their professional identity and status, helping to improve their work dignity and promote the enhancement of career recognition dignity. Thus, the following hypothesis is put forward in this study:

**H1a.**  *Perceived human–robot collaboration positively influences the perceived decent work of migrant workers.*

Perceived decent work enhances the well-being of migrant workers by fulfilling their survival, social connection, and self-determination needs. First, according to the theory of survival, relationships, and growth, survival needs are the most basic human needs. Perceived decent work means that migrant workers receive higher income and social security, which facilitates the fulfillment of their survival needs (Duffy et al., 2016), thereby improving their quality of life and enhancing their happiness. Second, the theory of survival, relationships, and growth posits that the need to maintain relationships with others is a fundamental human need, and social connections are essential for maintaining these relationships. Perceived decent work helps migrant workers build confidence in establishing connections with others, prompting them to form broader social connections with colleagues, friends, neighbors, and others in the city (Kozan et al., 2019), thereby meeting their social connection needs and increasing their happiness. Finally, the requirement for growth refers to an individual’s aspiration for personal development, corresponding to Maslow’s esteem and self-actualization needs. The essence of decent work requires workers to achieve growth in their jobs (ILO, 1999). On the one hand, perceived decent work fulfills migrant workers’ needs for growth and development, thereby enhancing their happiness; on the other hand, perceived decent work stimulates migrant workers’ job competence and sense of achievement, generating positive emotions from realizing their personal value, fulfilling their need for self-actualization (Duffy et al., 2019), and promoting an increase in happiness. Hence, this study advances the following hypothesis.

**H1b.**  *Perceived decent work positively influences the well-being of migrant workers.*

According to the resource conservation theory’s perspective on the resource gain spiral, an increase in resources can promote a further increase in resources (Hobfoll, 1989). Perceived decent work, by meeting migrant workers’ needs for survival, social connections, and growth and development, encourages greater work engagement, thereby further enhancing their individual resource reserves and promoting an increase in their well-being. In other words, perceived human–robot collaboration can improve migrant workers’ well-being by facilitating perceived decent work, with perceived decent work serving as a mediator between perceived human–robot collaboration and migrant workers’ well-being. As such, the present study posits the following hypothesis.

**H1c.**  *Perceived human–robot collaboration positively influences migrant workers’ well-being through the mediating role of perceived decent work.*

### 3.2. The Negative Effect of Perceived Human–Robot Collaboration on Migrant Workers’ Well-Being Through Job Replacement Anxiety

Job replacement anxiety is a form of anxiety caused by concerns about being replaced by robots or other artificial intelligence devices. This anxiety arises not only from real-world observations but also from a lack of information about the consequences of AI applications and inherent anxiety (J. Li & Huang, 2020). First, from the perspective of real-world experience, the use of robots in frontline jobs has significantly substituted repetitive tasks. For migrant workers, the phenomenon of robots replacing human labor in their environment generates concerns about the continuity of their work, leading to job replacement anxiety. Second, although robots have replaced certain jobs, they have also created new employment opportunities, and workers can achieve reemployment through job transitions, meaning human labor has not been massively displaced (Hamid et al., 2017). However, due to migrant workers’ limited understanding of the impact of robots, artificial intelligence, and other technologies, their perceptions of the consequences of AI applications are skewed, leading to the development of job replacement anxiety. Third, human–robot collaboration has raised the skill requirements for jobs. As migrant workers generally have lower educational levels and have been engaged in repetitive, low-skill jobs for a long time, they may experience greater difficulties and pressure when facing the higher skill demands imposed by human–robot collaboration. In such cases, the fear of being unable to meet the skill requirements for human–robot collaboration may result in higher levels of job replacement anxiety. Based on this, the following hypothesis is proposed:

**H2a.**  *Perceived human–robot collaboration positively influences job replacement anxiety.*

The conservation of resources theory posits that individuals who have suffered resource loss are less likely to participate in effective resource investment activities (Hobfoll et al., 2018). On one hand, greater job replacement anxiety signals that migrant workers worry about the possible loss of their employment. This anxiety impairs the social exchange relationship they have with employers, which in turn weakens the psychological contract, lowers work engagement, and adversely affects their well-being (Greenhalgh & Rosenblatt, 1984; Rosenblatt et al., 1999). On the other hand, migrant workers with higher job replacement anxiety are more likely to anticipate the loss of their jobs in the future. As a source of stress, concerns about unemployment or financial hardship make migrant workers feel tense and uneasy, diminishing their perceived well-being. For instance, Konuk et al. (2023) found that job replacement anxiety negatively affects employee well-being in their study of frontline service workers. In light of this, the following hypothesis is proposed:

**H2b.**  *Job replacement anxiety negatively influences migrant workers’ well-being.*

Research indicates that the impact of anticipated negative events on individuals may be greater than the impact of the events themselves (Lazarus & Folkman, 1984). According to the resource conservation theory’s perspective on the resource loss spiral, individuals experiencing resource loss struggle to engage in effective resource investment activities. This makes the initial resource loss of the individual lead to a further loss of resources (Hobfoll, 1989). The reduction in psychological resources in migrant workers caused by human–robot collaboration further diminishes their resource investment in work and reduces the likelihood of acquiring new resources, thereby weakening their well-being. In other words, perceived human–robot collaboration can reduce migrant workers’ well-being by triggering job replacement anxiety, with job replacement anxiety serving as a mediator between perceived human–robot collaboration and migrant workers’ well-being. On this basis, the hypothesis below is formulated:

**H2c.**  *Perceived human–robot collaboration negatively affects migrant workers’ well-being through the mediating role of job replacement anxiety.*

### 3.3. The Moderating Role of Perceived Organizational Support

According to the resource caravan and passage principles of the resource conservation theory, resources are situated within particular environments, where environmental conditions may either promote and cultivate resources or restrict and impede their growth (Hobfoll et al., 2018). A higher level of perceived organizational support can enhance organizational resources, thereby helping migrant workers acquire new resources and promoting resource gains. During human–robot collaboration, support provided by the organization, such as skill training, helps migrant workers improve work efficiency, reduce physical labor intensity, and enhance their work skills. This thereby reinforces the positive influence of perceived human–robot collaboration on perceived decent work. On the basis of this, the hypothesis that follows is put forward:

**H3a.**  *Perceived organizational support exerts a positive moderating influence on the association between perceived human–robot collaboration and perceived decent work.*

Resource conservation theory suggests that in the context of resource loss, the supplementation and increase in external resources become more valuable (Hobfoll et al., 2018). Migrant workers with higher perceived organizational support can receive more external resources, such as skill training, which helps alleviate pessimistic emotions. In response to the work demands triggered by human–robot collaboration, higher organizational support can replenish the resources consumed during the collaboration process. This is manifested in providing migrant workers with relevant skill training and appropriate job positions, thus mitigating job replacement anxiety caused by human–robot collaboration. For migrant workers with lower perceived organizational support, the lack of additional resources to offset the resource loss caused by human–robot collaboration makes them more prone to entering a resource loss spiral, thereby intensifying job replacement anxiety. Therefore, the hypothesis outlined below is formulated:

**H3b.**  *Perceived organizational support exerts a negative moderating effect on the relationship between perceived human–robot collaboration and job replacement anxiety.*

According to resource caravan and passage principles, as well as the resource gain spiral concept in resource conservation theory, environmental conditions are capable of exerting a nurturing and enriching function on the resources contained within them (Hobfoll et al., 2018). Migrant workers with higher perceived organizational support are more likely to invest additional resources in human–robot collaboration, which helps promote the development of the resource gain spiral. This, in turn, further enhances well-being by improving perceived decent work through human–robot collaboration. In contrast, migrant workers with lower perceived organizational support, due to the lack of environmental conditions that supplement their resources, are more inclined to conserve their limited resources (Halbesleben et al., 2014). As a result, they invest less in human–robot collaboration, reducing the likelihood of resource gains from such collaboration, weakening its positive effect on perceived decent work, and inhibiting improvements in well-being. Consequently, the hypothesis stated below is formulated:

**H3c.**  *Perceived organizational support positively moderates the mediating role of perceived decent work in the relationship between perceived human–robot collaboration and migrant workers’ well-being.*

According to the paradox of gain and the resource loss spiral in resource conservation theory, in situations of resource loss, the supplementation and increase in resources are of greater value (Hobfoll et al., 2018). Migrant workers with higher perceived organizational support have more external resource support, which reduces the likelihood of experiencing resource loss in human–robot collaboration. This, in turn, alleviates job replacement anxiety caused by perceived human–robot collaboration and helps slow the development of the resource loss spiral, thus diminishing its suppressive effect on well-being. For migrant workers with lower perceived organizational support, the lack of external resource supplementation may lead to more challenges and difficulties, resulting in emotional exhaustion. This will further increase their concerns about being unable to meet the skill requirements of their job positions, intensifying job replacement anxiety triggered by perceived human–robot collaboration. The increased job replacement anxiety will lead migrant workers into the resource loss spiral, amplifying the suppressive effect of perceived human–robot collaboration through job replacement anxiety on migrant workers’ well-being. Therefore, the hypothesis outlined below is formulated:

**H3d.**  *Perceived organizational support exerts a negative moderating influence on the mediating function of job replacement anxiety in the relationship linking perceived human–robot collaboration to migrant workers’ well-being.*

**Figure 1:**
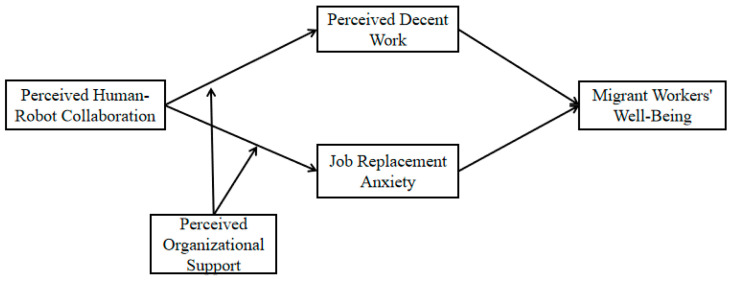
Theoretical model diagram.

Drawing on the above discussion, the theoretical model for this study is constructed and illustrated in Figure 1.

## 4. Methods

### 4.1. Data Collection and Sample Characteristics

This study adopts a two-stage survey method to collect data. Through a screening of their job positions, and adhering to the principle of random sampling, the enterprises selected for this study were determined. Based on a selection of the enterprises’ locations, industries, and basic characteristics, four industries were ultimately chosen, including electronics manufacturing, automobile manufacturing, food manufacturing, and apparel manufacturing. The sample consisted of 12 manufacturing enterprises, located in seven provinces: Zhejiang, Jiangsu, Guangdong, Shandong, Hebei, Liaoning, and Jilin.

Initially, migrant workers employed at the selected enterprises were entrusted to provide contact information for the human resources departments of their respective companies. Subsequently, direct consultations were conducted with the human resources department staff to discuss the research details. Upon obtaining their consent, the target respondents for the survey and the guidelines for completing the questionnaire were communicated to them. With the support of the human resources departments, the first phase of the survey was conducted in October 2024. All participants voluntarily agreed to take part in this survey.

In all, 550 questionnaires were distributed and obtained from migrant workers on the frontline through work groups and other means. After screening and organizing, a total of 445 valid questionnaires were secured, resulting in an effective response rate of 80.9%. The second phase of the survey was carried out two weeks later, targeting the respondents who completed the 445 valid questionnaires in the first phase. A second round of questionnaires was distributed, resulting in the collection of 410 questionnaires, with a sample attrition rate of 7.87%. Finally, after excluding questionnaires with irregular answers and those failing the attention check, a total of 382 valid questionnaires were preserved. Consequently, the final effective response rate stands at 93.17%.

Among the respondents, 60.995% identified as male, while 39.005% identified as female. In terms of age distribution, 13.351% were under 25 years old, 42.408% fell into the 25–34 age group, 36.126% were aged 35–44, and 8.115% were 45 years old or older. With respect to educational attainment, 38.743% had an education level of junior high school or below, 42.670% held a high school education (encompassing secondary vocational school, technical school, and vocational high school qualifications), and 18.586% possessed a college degree or higher.

### 4.2. Variable Measurement

All core variables in this study were measured using scales that are widely applied and well-validated both domestically and internationally. These scales underwent revision through back-translation and were proofread by two management experts with proficient English skills, which ensured the accuracy and clarity of the measurement items. A five-point Likert scale was adopted for scoring: 1 indicated “Strongly Disagree,” and 5 indicated “Strongly Agree.”

Well-being. The assessment of migrant workers’ well-being in this research was adapted from the employee well-being scale. This scale was originally developed by Zheng et al. (2015) to fit the Chinese context. Representative items from the scale include “Overall, I feel positive about myself and am satisfied with the outcomes of past events.” For this scale, the Cronbach’s α coefficient is 0.951.

Perceived human–robot collaboration (HRC). Perceived human–robot collaboration was measured using the scale proposed by G. He et al. (2025), which consists of three items. Representative items include “I collaborate with robots to solve work-related problems and challenges.” For this scale, the Cronbach’s α coefficient is 0.869.

Perceived decent work (PDW). The measurement of perceived decent work in this study is based on four dimensions: wage income, working hours, career development, and occupational recognition, drawing from established scales both domestically and internationally. The scale contains a total of 13 items. The wage income dimension is based on the fair compensation subscale from Ferraro et al.’s (2018) perceived decent work scale, consisting of four items. The working hours dimension is derived from the work–life balance subscale from D. Liu and Xu’s (2017) perceived decent work scale, which includes aspects such as overtime frequency and working hours, containing three items. The career development dimension is based on the personal career development section of the meaningful and productive replacement scale from Ferraro et al. (2018), which includes three items. The occupational recognition dimension comes from the occupational recognition and dignity subscale in Mao et al.’s (2014) perceived decent work scale, containing three items. Representative items include “The income I receive from my job enables me to live with dignity and autonomy,” “My average daily working hours are acceptable,” and “My work is helpful for my future career development.” For this scale, the Cronbach’s α coefficient is 0.909.

Job replacement anxiety (JRA). In this study, job replacement anxiety among migrant workers due to human–robot collaboration is measured using the job replacement anxiety subscale from the Artificial Intelligence Anxiety Scale developed by J. Li and Huang (2020). The term “artificial intelligence” was replaced with “robot” to better suit the context of this study. The scale includes three items, with representative questions such as “I worry that robots will replace my job in the future.” For this scale, the Cronbach’s α coefficient is 0.884.

Perceived organizational support (POS). The perceived organizational support of migrant workers is measured using a two-dimensional scale developed by McMillan (1997). Representative items include “The organization cares about my welfare” and “The organization provides me with a good working environment and facilities.” The Cronbach’s α for this scale is 0.909.

Control variables. Given that individual characteristics, organizational characteristics, and robot characteristics may all influence the effectiveness of perceived human–robot collaboration, this study controls for these variables. Individual characteristics encompass the respondent’s gender (coded as 1 for male and 0 for female), age (coded as 1 for 25 years old or younger, 2 for 26–35 years old, 3 for 36–45 years old, and 4 for 46 years old or older), educational background (coded as 1 for middle school or below, 2 for high school, 3 for vocational school or technical school, and 4 for college or higher), and duration of experience with human–robot collaboration (coded as 1 for less than six months, 2 for six months to one year, 3 for one to three years, and 4 for more than three years). Furthermore, to control for the influence of urban integration on migrant workers’ well-being, this study also includes their adaptability to urban life as a control variable, which is measured using a five-point Likert scale (ranging from 1 = very unadapted to 5 = very adapted). Considering that migrant workers frequently change jobs, this study also controls for the number of jobs held by the migrant workers (fewer than 3 = 1; 3 to 5 = 2; more than 5 = 3). To address the potential impact of managerial roles on well-being, this study controls for the migrant workers’ job position (manager = 1; general employee = 0). Organizational characteristics include firm size (fewer than 100 employees = 1; 100 to 500 employees = 2; more than 500 employees = 3) and ownership type (state-owned enterprise = 1; private enterprise = 0). Given the potential influence of industry and regional characteristics on migrant workers’ well-being, this study also controls for the industry and region of the enterprise. Furthermore, to control for the differences in robot characteristics’ impact on migrant workers’ well-being, this study controls for robot size (small = 1; medium = 2; large = 3) and robot failure rate (very high = 1; relatively high = 2; average = 3; relatively low = 4; very low = 5).

## 5. Results

### 5.1. Confirmatory Factor Analysis

This research utilizes AMOS 26.0 software to perform Confirmatory Factor Analysis (CFA), aiming to examine whether the measurement validity of each variable meets the specified criteria. Prior to testing for convergent and discriminant validity, it is necessary to examine the model fit of the variables. Given that an excessive number of items in the scale may reduce the ratio of sample size to estimated parameters, this study bundles multidimensional variables for analysis. Additionally, since CFA aims to test the distinctiveness between core variables rather than exploring the interrelationships among individual items, prior research has also suggested that bundling variables with many items is a reasonable approach. Specifically, this study bundles variables according to their dimensions, namely, migrant workers’ well-being into three bundles, perceived decent work into four bundles and perceived organizational support into two bundles. Subsequently, five distinct constructs—migrant workers’ well-being, perceived human–robot collaboration, perceived decent work, job replacement anxiety and perceived organizational support—are proposed as a five-factor model. Model fit is subsequently assessed by comparing fit indices across different construct aggregation approaches. Table 1 shows that the fit indices of the five-factor model meet the required standards and are more favorable.

### 5.2. Descriptive Statistics and Correlation Analysis

The results of descriptive statistics and correlation analysis for the core variables in this research are presented in Table 2. It can be noted that the correlation coefficients among all variables are below 0.6, which suggests the absence of high correlation between the variables.

### 5.3. Common Method Bias Test

While this study adopted a multi-wave survey approach to mitigate the influence of common method bias, all data required for the research were collected via self-administered questionnaires from participants. This makes it challenging to fully eliminate the occurrence of common method bias. This study employs Harman’s single-factor test to address this issue. The results indicated that the unrotated first factor explained 31.166% of the total variance, which is below the critical threshold of 40%. This suggests that the issue of common method bias in this study is not prominent, enabling the continuation of further analyses.

### 5.4. Hypothesis Testing

#### 5.4.1. Regression Analysis

To investigate the effects of perceived human–robot collaboration on perceived decent work and job replacement anxiety, as well as the impacts of perceived decent work and job replacement anxiety on migrant workers’ well-being, hierarchical regression analysis was utilized. The outcomes of this regression analysis are presented in Table 3. The results clearly demonstrate that perceived human–robot collaboration has a positive effect on perceived decent work (β = 0.244, *p* < 0.01) and also positively impacts job replacement anxiety (β = 0.116, *p* < 0.05). Furthermore, perceived decent work exerts a positive influence on migrant workers’ well-being (β = 0.554, *p* < 0.01), whereas job replacement anxiety has a negative effect on their well-being (β = −0.309, *p* < 0.01). As a result, hypotheses H1a, H1b, H2a, and H2b are all validated.

#### 5.4.2. Empirical Test of the Mediating Roles of Perceived Decent Work and Work Replacement Anxiety

To test the mediating roles of perceived decent work and job replacement anxiety, this study employed Model 4 of the PROCESS 3.4 plugin, with the number of Bootstrap resamples set to 5000. The findings are presented in Table 4. As indicated, the mediating effect of perceived decent work was 0.110, and its 95% confidence interval was [0.067, 0.160]. Since this interval does not include 0, it confirms that perceived decent work exerts a significant mediating effect. Therefore, hypothesis H1c is supported. Likewise, the mediating effect of job replacement anxiety was −0.043, with a 95% confidence interval of [−0.078, −0.010], which also excludes 0. This result demonstrates that job replacement anxiety also has a significant mediating effect. Therefore, hypothesis H1d is supported.

#### 5.4.3. Empirical Test of the Moderating Effects of Perceived Organizational Support

To investigate the moderating roles of perceived organizational support in the relationships between perceived human–robot collaboration and perceived decent work, as well as between perceived human–robot collaboration and job replacement anxiety, hierarchical regression analysis was utilized. The results are presented in Table 5. The data indicate that the interaction term between perceived human–robot collaboration and perceived organizational support shows a positive effect on perceived decent work (β = 0.102, *p* < 0.05), while this interaction term negatively affects job replacement anxiety (β = −0.160, *p* < 0.05). Thus, hypotheses H3a and H3b are both supported.

**Figure 2:**
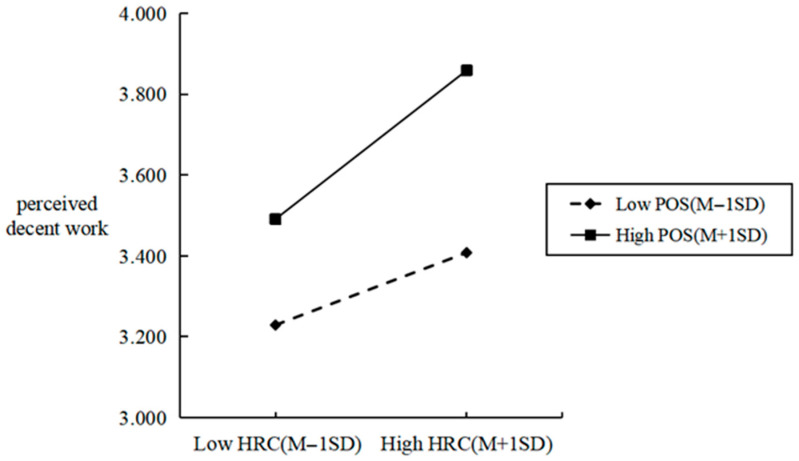
Moderating effect of perceived organizational support on the relationship between perceived human–robot collaboration and perceived decent work.

**Figure 3:**
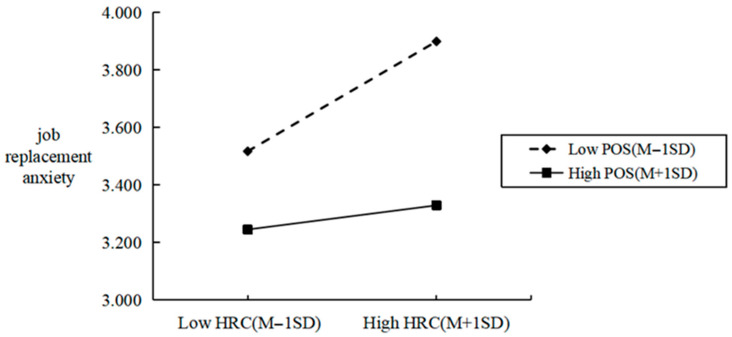
Moderating effect of perceived organizational support on the relationship between perceived human–robot collaboration and job replacement anxiety.

Figure 2 and Figure 3 illustrate the moderating effects of perceived organizational support. It can be observed that, in comparison to scenarios with low perceived organizational support, the slope of the relationship between perceived human–robot collaboration and perceived decent work is steeper under conditions of high perceived organizational support. Conversely, the slope linking perceived human–robot collaboration to job replacement anxiety is flatter in such high-support contexts. These findings further validate the previous research conclusions.

#### 5.4.4. Empirical Test of the Moderated Mediation Effect

To investigate the moderating role of perceived organizational support in the mediating effects of perceived decent work and job replacement anxiety, a conditional process model analysis was performed using Model 7 of the PROCESS 3.4 plugin, with 5000 bootstrap samples. The test results are displayed in Table 6. For the pathway through which perceived human–robot collaboration influences migrant workers’ well-being via perceived decent work: under conditions of low perceived organizational support (M − 1SD), the indirect effect of perceived human–robot collaboration on migrant workers’ well-being through perceived decent work is 0.058. Its 95% confidence interval is [0.013, 0.098], which excludes zero, indicating a significant indirect effect. When perceived organizational support is high (M + 1SD), the indirect effect of perceived human–robot collaboration on migrant workers’ well-being via perceived decent work is 0.120, with a 95% confidence interval of [0.067, 0.177] that also excludes zero, confirming this indirect effect is significant. The between-group difference is 0.062, with a 95% confidence interval of [0.007, 0.128] that excludes zero, signaling a significant difference. Additionally, the index statistic from the PROCESS 3.4 analysis reveals that the moderating effect of perceived organizational support on the indirect impact of perceived human–robot collaboration—via perceived decent work—on migrant workers’ well-being is 0.046. Its 95% confidence interval is [0.005, 0.095], which excludes zero, demonstrating a significant moderated mediation effect. In summary, the moderating function of perceived organizational support in the mediating role of perceived decent work within the association between perceived human–robot collaboration and migrant workers’ well-being is statistically significant, thereby supporting hypothesis H3c.

Regarding the pathway through which perceived human–robot collaboration affects migrant workers’ well-being via job replacement anxiety: under conditions of low perceived organizational support (M − 1SD), the indirect effect of perceived human–robot collaboration on migrant workers’ well-being through job replacement anxiety is −0.103. Its 95% confidence interval is [−0.155, −0.057], which excludes zero, indicating a significant indirect effect. When perceived organizational support is high (M + 1SD), the indirect effect of perceived human–robot collaboration on migrant workers’ well-being via job replacement anxiety is −0.023, with a 95% confidence interval of [−0.071, 0.024] that includes zero. This suggests that the indirect effect is not significant. The difference between the two groups stands at 0.080, accompanied by a 95% confidence interval of [0.020, 0.147] that excludes zero, confirming a significant difference. Additionally, the index statistic obtained from the PROCESS 3.4 analysis reveals that the moderating effect of perceived organizational support on the indirect impact of perceived human–robot collaboration—via job replacement anxiety—on migrant workers’ well-being is 0.060. Its 95% confidence interval is [0.015, 0.110], which excludes zero, demonstrating a significant moderated mediation effect, thereby supporting hypothesis H3d.

**Figure 4:**
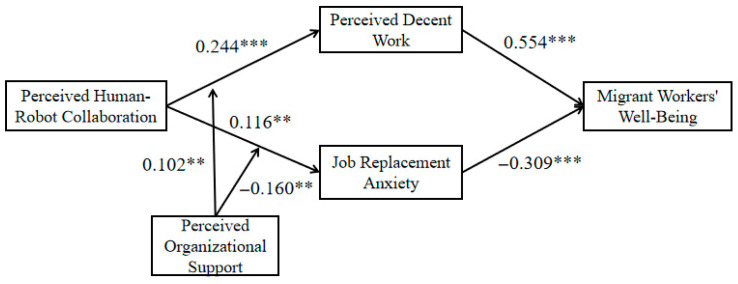
Overall regression results. *** *p* < 0.01, ** *p* < 0.05.

The main regression results of this study are shown in Figure 4.

## 6. Discussion

### 6.1. Theoretical Contributions

First, this study extends research on the antecedents of migrant workers’ well-being to the emerging work context of human–robot collaboration, offering a new perspective for future studies on these antecedents of migrant workers’ well-being. Existing studies on the impact of work characteristics on migrant workers’ well-being mostly explore traditional contexts, investigating factors such as income, working hours, job security, leadership characteristics, organizational climate, and organizational support (Zhou et al., 2025; Yu et al., 2019). However, there is a lack of research on the novel work characteristics of the digital intelligence era. Focusing on the expanding application of robots in the workplace, this study introduces human–robot collaboration into the analysis framework of factors influencing migrant workers’ well-being, broadening the context of antecedent research on migrant workers’ well-being and providing a new theoretical perspective for studying this topic in the digital intelligence era.

Second, this study verifies that perceived human–robot collaboration exerts both positive and negative impacts on migrant workers’ well-being, thereby reconciling the debate surrounding the influence of human–robot collaboration on employees’ well-being. Existing research on how human–robot collaboration affects employees’ well-being has yielded contradictory findings: some document positive effects, while others identify negative ones (Shaikh et al., 2023; Tang et al., 2023; N. C. Liu et al., 2023). By accounting for both the positive and negative impacts of perceived human–robot collaboration on migrant workers’ well-being, this study constructs a dual-edged sword effect model. This model addresses the issue through the dual lenses of resource gain and resource loss triggered by perceived human–robot collaboration, providing a balanced perspective on the ongoing debate of how perceived human–robot collaboration influences migrant workers’ well-being.

Third, this study reveals the mechanisms and boundary conditions of perceived human–robot collaboration’s influence on migrant workers’ well-being. Existing research primarily approaches this issue from the general employee perspective, analyzing the mechanisms through demand satisfaction, technological stress, and loneliness (Yang & Gao, 2025; Tang et al., 2023; N. C. Liu et al., 2023), and explores the contextual factors of human–robot collaboration’s impact on employee well-being from the angles of skill flexibility and attachment anxiety (Tang et al., 2023; N. C. Liu et al., 2023). This study, however, focuses on the real challenges faced by migrant workers, including limited human capital and low job quality. It uncovers the pathways through which perceived human–robot collaboration affects migrant workers’ well-being from the perspectives of perceived decent work and work replacement anxiety. Furthermore, this study emphasizes the pivotal role of perceived organizational support in enabling high-quality work among migrant workers, while identifying their moderating functions in the process through which perceived human–robot collaboration influences migrant workers’ well-being. Specifically, this research uncovers the underlying mechanisms driving the impact of perceived human–robot collaboration on migrant workers’ well-being and clarifies the boundary conditions of this effect. This study develops a theoretical framework to comprehend how perceived human–robot collaboration influences migrant workers’ well-being. It also provides practical implications for enhancing the positive effects of perceived human–robot collaboration on their well-being and mitigating its adverse outcomes.

### 6.2. Practical Implications

The policy implications derived from this study are as follows: First, the government should introduce a comprehensive set of policies that encourage enterprises to conditionally promote human–robot collaboration while strengthening regulation of robot applications. This would prevent large-scale automation from having a negative impact on migrant workers’ employment. At the same time, businesses should recognize the positive impact of human–robot collaboration on migrant workers’ decent labor and well-being, and actively explore ways to enhance migrant workers’ decent labor and well-being through human–robot collaboration in their management practices. On one hand, human–robot collaboration should be prioritized in hazardous job positions to relieve migrant workers from dangerous tasks, enabling perceived decent work and enhancing their well-being. On the other hand, human–robot collaboration should be conditionally expanded in repetitive positions to increase job diversity, improve work skills and efficiency, facilitate migrant workers’ career development and recognition, thereby enhancing their decent labor and well-being.

Second, there should be strengthened job security for migrant workers to prevent high levels of work replacement anxiety. Enterprises should provide targeted vocational training, such as course lectures, hands-on practice, and experience exchanges, to improve migrant workers’ skills in collaborating with robots and reduce work replacement anxiety caused by insufficient skills. Additionally, enterprises can implement special support programs for migrant workers to alleviate anxiety generated by large-scale automation. Enterprises should implement structural job redesign as a concrete management measure to match the application of robots and lower migrant workers’ job replacement anxiety. By redefining job contents, optimizing workflow, and clarifying the division of labor between humans and machines, enterprises can enhance the irreplaceability of migrant workers, so as to alleviate their psychological concerns and reduce their anxiety about job replacement.

Finally, enterprises should enhance support for migrant workers and strengthen their sense of organizational support. First, enterprises should actively listen to migrant workers’ needs and feedback through regular surveys and feedback mechanisms to understand the difficulties they face in their work and daily lives and make timely responses and adjustments. Second, enterprises should invest in skill training for migrant workers, employing supportive human resource management practices to enhance their work capabilities and facilitate personal career growth. Third, enterprises should provide migrant workers with good working conditions and facilities. In environments prone to noise, dust, and other harmful elements, the installation of noise reduction equipment, air purifiers, and similar facilities can help migrant workers feel that the enterprise values and invests in their work, thereby strengthening their sense of organizational support and promoting their well-being.

### 6.3. Limitations and Future Research Directions

This study specifically uncovers the dual-edged impact of perceived human–robot collaboration on migrant workers’ well-being; however, it has certain limitations that deserve further attention in future research. First, the perceived human–robot collaboration scale employed in this research assesses the degree to which migrant workers collaborate with robots, and the influence of such collaboration on their well-being was examined based on this measurement. Future studies could categorize different forms of human–robot collaboration—such as leading or following roles (Pasparakis et al., 2023)—and incorporate these into the research framework. This would help provide a more comprehensive and detailed understanding of how perceived human–robot collaboration affects migrant workers’ well-being.

Second, the moderating variables in this study are based on individual perception, which, while helping to avoid cross-level issues in the research, may introduce a degree of subjectivity. Future research could focus on relatively objective variables within the enterprise, such as the actual training provided or the number of specific support measures implemented by the company, and use these as moderating variables.

Additionally, the design characteristics of human–robot collaboration, such as robot design and workplace design, also influence the effectiveness of human–robot collaboration (Baltrusch et al., 2022). Future studies could incorporate robot characteristics, such as intelligence and safety, into the research framework to further investigate how these features modulate the effects of human–robot collaboration.

Finally, this study made use of a two-stage survey method for data collection to mitigate the issue of common method bias in sample sources to some extent. However, the results measured remain static. Future research could adopt longitudinal surveys, tracking migrant workers’ psychological perceptions over multiple time points as they collaborate with robots, in order to capture the changes in their perceptions during different stages of collaboration.

## 7. Conclusions

Drawing on resource conservation theory, this research constructed a dual-edged sword effect model to investigate the influence of perceived human–robot collaboration on migrant workers’ well-being. Through an empirical analysis of data from a two-stage survey of 382 migrant workers employed in manufacturing enterprises, the following conclusions were drawn: ① Perceived human–robot collaboration can both positively influence migrant workers’ well-being by enhancing perceived decent labor, and negatively affect their well-being by increasing job replacement anxiety. ② Perceived organizational support both plays a positive moderating role in the relationship between perceived human–robot collaboration and perceived decent work, as well as in the mediating function of perceived decent work within the connection between perceived human–robot collaboration and migrant workers’ well-being. ③ Perceived organizational support exerts a negative moderating influence on two aspects: the relationship between perceived human–robot collaboration and job replacement anxiety, and the mediating function of job replacement anxiety in the association linking perceived human–robot collaboration to migrant workers’ well-being.

## Figures and Tables

**Table 1:** Confirmatory Factor Analysis results.

Model	χ^2^	df	χ^2^/df	RMR	TLI	CFI	RMSEA
Five-factor (A, B, C, D, E)	668.733	254	2.633	0.036	0.928	0.939	0.065
Four-factor (A + B, C, D, E)	1403.784	265	5.297	0.083	0.813	0.835	0.106
There-factor (A, B + C + D, E)	1921.193	269	7.142	0.059	0.733	0.760	0.127
Two-factor (A, B + C + D + E)	2788.483	274	10.177	0.079	0.601	0.635	0.155
One-factor (A + B + C + D + E)	3982.348	275	14.481	0.088	0.414	0.462	0.188

A = perceived human–robot collaboration; B = migrant workers’ well-being; C = perceived decent work; D = job replacement anxiety; E = perceived organizational support. RMR = Root Mean Square Residual, TLI = Tucker–Lewis Index, CFI = Comparative Fit Index, RMSEA = Root Mean Square Error of Approximation.

**Table 2:** Descriptive statistics and correlation analysis results.

Variables	1	2	3	4	5
1. Migrant Workers’ Well-Being	(0.762)				
2. Perceived Human–Robot Collaboration	0.436 ***	(0.832)			
3. Perceived Decent Work	0.525 ***	0.315 ***	(0.787)		
4. Job Replacement Anxiety	−0.317 ***	0.122 **	−0.186 ***	(0.850)	
5. Perceived Organizational Support	0.534 ***	0.226 ***	0.380 ***	−0.327 ***	(0.780)
M	3.282	3.442	3.508	3.481	3.223
SD	0.661	0.693	0.564	0.600	0.670

The square root of AVE is in parentheses. M = mean, SD = standard deviation. *** *p* < 0.01, ** *p* < 0.05.

**Table 3:** Regression analysis results.

Variables	Perceived Decent Work		Job Replacement Anxiety		Well-Being		
	Model 1	Model 2	Model 3	Model 4	Model 5	Model 6	Model 7
Gender	0.041	0.022	0.080	0.071	0.010	−0.013	0.035
Age	−0.049 *	−0.044	0.066 **	0.068 **	−0.058 *	−0.031	−0.038
Educational background	0.011	0.027	−0.058 *	−0.050	0.016	0.010	−0.002
Industry	0.032	0.009	0.028	0.017	0.066 **	0.049 *	0.075 ***
Ownership type	−0.050	−0.069	0.012	0.002	−0.078	−0.050	−0.074
Firm size	0.113 ***	0.126 ***	−0.066	−0.060	0.064	0.001	0.043
Duration of experience with HRC	0.063 **	0.063 **	−0.073 **	−0.073 **	0.027	−0.008	0.004
Robot size	−0.017	−0.016	0.091 **	0.092 **	0.014	0.023	0.042
Robot failure rate	−0.035	−0.038	0.029	0.028	−0.034	−0.015	−0.025
province	0.037 **	0.027 *	−0.011	−0.015	0.053 ***	0.033 **	0.049 ***
Urban adaptability	0.050 *	0.029	−0.063 **	−0.073 **	0.180 ***	0.152 ***	0.160 ***
Number of jobs	0.012	0.017	−0.003	−0.001	0.003	−0.004	0.002
Job position	0.052	0.061	0.043	0.048	−0.037	−0.066	−0.024
HRC		0.244 ***		0.116 **			
PDW						0.554 ***	
JRA							−0.309 ***
R^2^	0.097	0.181	0.067	0.084	0.155	0.357	0.229
ΔR^2^		0.084		0.017		0.202	0.074
F	3.024	5.791	2.027	2.393	5.200	14.565	7.765

HRC = perceived human–robot collaboration; PDW = perceived decent work; JRA = job replacement anxiety. *** *p* < 0.01, ** *p* < 0.05, * *p* < 0.1.

**Table 4:** Mediating effect test results.

Mediator Variable	Effect	Boot SE	Boot LLCI	Boot ULCI
Perceived Decent Work	0.110	0.024	0.067	0.160
Job Replacement Anxiety	−0.043	0.017	−0.078	−0.010

**Table 5:** Empirical test results of the moderating effect of perceived organizational support.

Variables	Perceived Decent Work			Job Replacement Anxiety		
	Model 1	Model 2	Model 3	Model 4	Model 5	Model 6
Gender	0.022	0.047	0.056	0.071	0.042	0.029
Age	−0.044	−0.028	−0.025	0.068 **	0.050 *	0.046
Educational background	0.027	0.026	0.029	−0.050	−0.050	−0.053 *
Industry	0.009	0.019	0.019	0.017	0.005	0.007
Ownership type	−0.069	−0.042	−0.036	0.002	−0.029	−0.039
Firm size	0.126 ***	0.117 ***	0.119 ***	−0.060	−0.049	−0.053
Duration of experience with HRC	0.063 **	0.049 **	0.047 *	−0.073 **	−0.057 **	−0.053 *
Robot size	−0.016	−0.015	−0.015	0.092 **	0.091 **	0.090 **
Robot failure rate	−0.038	−0.045	−0.049	0.028	0.037	0.043
province	0.027 *	0.025 *	0.024 *	−0.015	−0.012	−0.010
Urban adaptability	0.029	0.006	−0.002	−0.073 **	−0.046	−0.040
Number of jobs	0.017	−0.004	−0.005	−0.001	0.025	0.026
Job position	0.061	0.079	0.079	0.048	0.026	0.027
HRC	0.244 ***	0.188 ***	−0.133	0.116 **	0.183 ***	0.684 ***
POS		0.264 ***	−0.087		−0.313 ***	0.237
HRC × POS			0.102 **			−0.160 **
R^2^	0.181	0.269	0.277	0.084	0.192	0.209
ΔR^2^		0.088	0.008		0.108	0.017
F	5.791	8.957	8.716	2.393	5.791	6.030

HRC = perceived human–robot collaboration; POS = perceived organizational support. *** *p* < 0.01, ** *p* < 0.05, * *p* < 0.1.

**Table 6:** Empirical test results of the moderated mediation effect of perceived organizational support.

Mediator Variable	Level	Effect	Boot SE	Boot LLCI	Boot ULCI
Perceived Decent Work	Low (M − 1SD)	0.058	0.022	0.013	0.098
	High (M + 1SD)	0.120	0.028	0.067	0.177
	Group Difference	0.062	0.031	0.007	0.128
Job Replacement Anxiety	Low (M − 1SD)	−0.103	0.025	−0.155	−0.057
	High (M + 1SD)	−0.023	0.024	−0.071	0.024
	Group Difference	0.080	0.033	0.020	0.147

## Data Availability

The data that support the findings of this study are available upon request from the corresponding authors. The data are not publicly available due to privacy or ethical restrictions.
